# Synthesis and Structure Insights of Two Novel Broad-Spectrum Antibacterial Candidates Based on (*E*)-*N*′-[(Heteroaryl)methylene]adamantane-1-carbohydrazides

**DOI:** 10.3390/molecules25081934

**Published:** 2020-04-22

**Authors:** Lamya H. Al-Wahaibi, Natalia Alvarez, Olivier Blacque, Nicolás Veiga, Aamal A. Al-Mutairi, Ali A. El-Emam

**Affiliations:** 1 Department of Chemistry, College of Sciences, Princess Nourah bint Abdulrahman University, Riyadh 11671, Saudi Arabia; lhalwahaibi@pnu.edu.sa; 2 Química Inorgánica, Facultad de Química, Universidad de la República, Av. General Flores 2124, Montevideo 11800, Uruguay; nalvarez@fq.edu.uy (N.A.); nveiga@fq.edu.uy (N.V.); 3 Department of Chemistry, University of Zurich, Winterthurerstrasse 190, 8057 Zurich, Switzerland; 4 Department of Chemistry, College of Sciences, Al Imam Mohammad Ibn Saud Islamic University (IMSIU), Riyadh 11623, Saudi Arabia; aamutairi@imamu.edu.sa; 5 Department of Medicinal Chemistry, Faculty of Pharmacy, Mansoura University, Mansoura 35516, Egypt

**Keywords:** adamantane-1-carbohydrazides, antibacterial activity, crystal structure, DFT, Hirshfeld surface analysis, IR, UV-Vis spectra

## Abstract

Two new *N*′-heteroarylidene-1-carbohydrazide derivatives, namely; *E*-*N*′-[(pyridine-3-yl)methylidene]adamantane-1-carbohydrazide (**1**) and *E*-*N*′-[(5-nitrothiophen-2-yl)methylidene]adamantane-1-carbohydrazide (**2**), were produced via condensation of adamantane-1-carbohydrazide with the appropriate heterocyclic aldehyde. Both compounds were chemically and structurally characterized by ^1^H-NMR, ^13^C-NMR, infrared and UV-vis spectroscopies, and single crystal X-ray diffraction. The study was complemented with density functional theory calculations (DFT). The results show an asymmetrical charge distribution in both compounds, with the electron density accumulated around the nitrogen and oxygen atoms, leaving the positive charge surrounding the N-H and C-H bonds in the hydrazine group. Consequently, the molecules stack in an antiparallel fashion in the crystalline state, although the contribution of the polar contacts to the stability of the lattice is different for **1** (18%) and **2** (42%). This difference affects the density and symmetry of their crystal structures. Both molecules show intense UV-Vis light absorption in the range 200–350 nm (**1**) and 200–500 nm (**2**), brought about by π → π* electronic transitions. The electron density difference maps (EDDM) revealed that during light absorption, the electron density flows within the π-delocalized system, among the pyridyl/thiophene ring, the nitro group, and the *N*′-methyleneacetohydrazide moiety. Interestingly, compounds **1** and **2** constitute broad-spectrum antibacterial candidates, displaying potent antibacterial activity with minimal inhibitory concentration (MIC) values around 0.5–2.0 μg/mL. They also show weak or moderate antifungal activity against the yeast-like pathogenic fungus *Candida albicans*.

## 1. Introduction

Heterocyclic carboxylic acid hydrazides and their *N*′-arylidene derivatives were early identified as potent chemotherapeutic agents for the control of mycobacterial infections [1,2]. As a result of extensive research based on homocyclic and heterocyclic hydrazides and their *N*′-arylidene derivatives, numerous derivatives were developed and proved to be superior to their prototype drugs [3,4,5,6,7,8]. In addition, various hydrazide and hydrazine analogues were proved to possess potent antibacterial and antifungal [9,10,11,12], antiviral [13], anticancer [14,15,16], and antileishmanial [17,18] activities. Moreover, adamantane derivatives have long been known for their diverse biological activities and several adamantane-based drugs are currently used as efficient therapeutic agents for the treatment of various pathological disorders [19,20,21]. Moreover, adamantane-1-carbohydrazide and hydrazone derivatives were previously studied and displayed potent antibacterial and antifungal activities [22,23,24,25].

In view of the above-mentioned observations and continuing with recent studies on the chemical and pharmacological properties of adamantane-based derivatives [26,27,28,29], we report herein the synthesis of two novel *N*′-[(heteroaryl)methylene]adamantane-1-carbohydrazides that display potent antibacterial activity. Both compounds were chemically and structurally characterized using a wide range of experimental and computational techniques, including ^1^H-NMR, ^13^C-NMR, infrared and UV-vis spectroscopies, single crystal X-ray diffraction, and density functional theory calculations (DFT) calculations. The results allowed us to gain insights into the chemical identity, molecular structure, solid-state intermolecular interactions, and light absorption of these two new broad-spectrum antibacterial compounds.

## 2. Results and Discussion

### 2.1. Chemical Synthesis

**Scheme 1:**
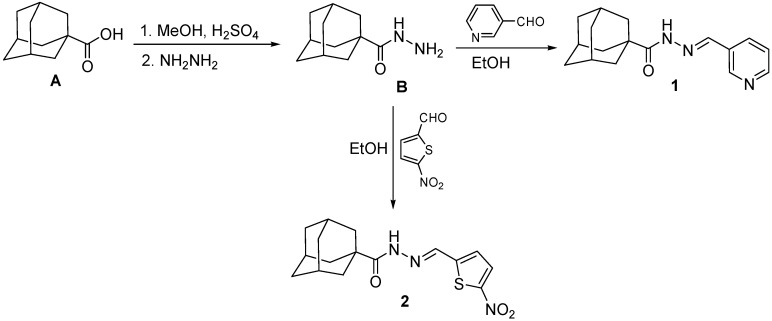
Synthesis of compounds **1** and **2**.

The (*E*)-*N*′-[(heteroaryl)methylene]adamantane-1-carbohydrazides **1** and **2** were prepared starting with adamantane-1-carboxylic acid **A** via esterification with methanol to yield the methyl ester, which was subsequently reacted with hydrazine to yield adamantane-1-carbohydrazide **B** [30]. The hydrazide **B** was then reacted with pyridine-3-carboxaldehyde or 5-nitrothiophene- 2-carboxaldehyde in ethanol to yield the target compounds **1** and **2** in 82% and 95% yields, respectively (Scheme 1). The ^1^H-NMR spectra of compounds **1** and **2** were in full agreement with their structures. The NH protons were shown as singlets at δ 11.01 and 11.24 and the CH=N protons at δ 8.81 and 8.68 ppm, respectively. The adamantyl CH and CH_2_ protons (15H) were shown as three distinguished peaks at δ 2.03, 2.02 (3H), 1.88, 1.87 (6H), and 1.72, 1.68–1.73 ppm (6H). In addition, the integrations and multiplicities of the pyridine and thiophene protons were properly shown. The ^13^C-NMR spectra exhibited the C=O carbons at δ 173.86 and 174.18 ppm and the CH=N carbons at δ 144.35 and 140.63 ppm, respectively. The adamantyl carbons were shown as four distinguished peaks. The pyridine and thiophene carbons were shown in the expected regions.

### 2.2. Crystal Structures

**Figure 1:**
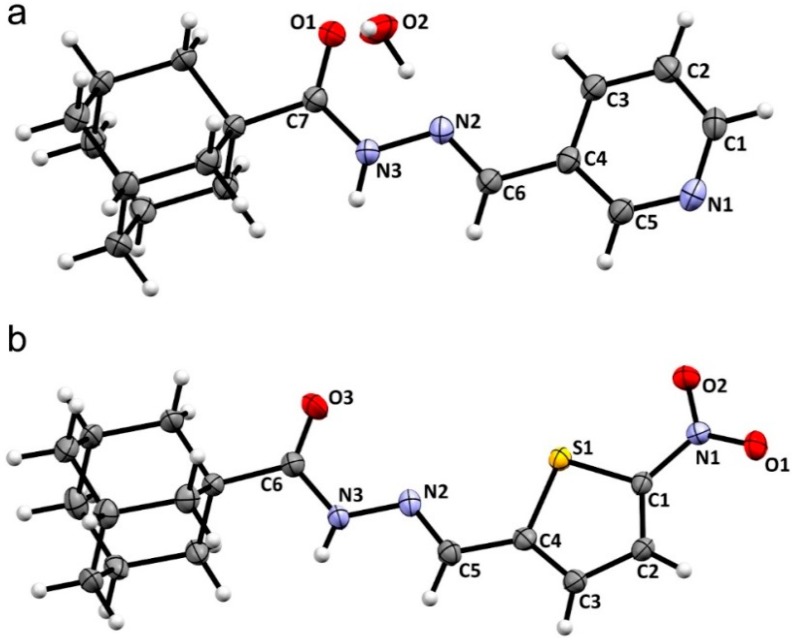
ORTEP diagrams at 50% probability for the asymmetric units of **1** (**a**) and **2** (**b**) with atom numbering scheme.

Single crystal X-ray diffraction was used to determine the crystal structures of compounds **1** and **2**. A summary of the crystallographic data and structure refinement parameters are listed in Table 1. The Oak Ridge Thermal Ellipsoid Plot (ORTEP) representation at 50% probability corresponding to the asymmetric units of **1** and **2** are depicted in Figure 1.

**Figure 2:**
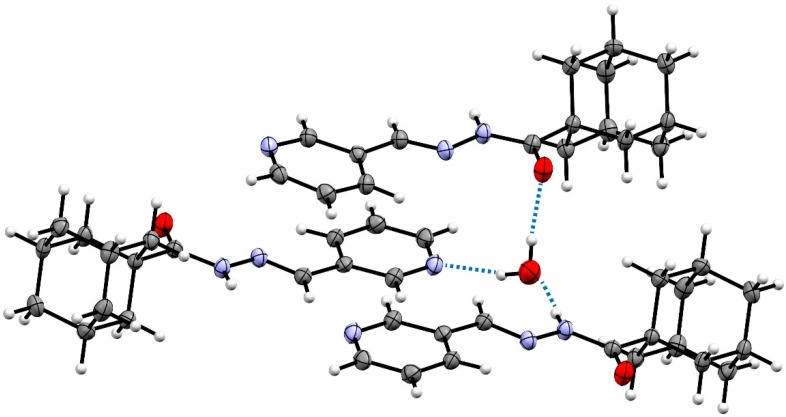
Hydrogen bond (blue dashed line) scheme for compound **1**. Atom color code: C (grey), H (white), O (red), N (blue), S (yellow).

The water molecule in the crystal structure of **1** participates in three classical hydrogen bonds, two as a donor, O-H···O(carbonyl) and O-H···N(pyridine), and one acting as an acceptor, N2-H···O (Table 2, Figure 2). Additionally, O1 participates in a non-classical hydrogen bond C2-H2···O1 with an H2···O1 distance of 2.46 Å and an angle of 129.20°. On the other hand, compound **2** presents only one classical hydrogen bond involving the N atom of the hydrazide group and an O atom of the nitro group of a contiguous molecule (Table 2). Also, the O3 (carbonyl) participates in a non-classical hydrogen bond C2-H2···O3 with an H2···O3 distance of 2.80 Å and an angle of 126.62°. The participation of the carbonyl O atoms in low-energy H-bonds was evidenced by the shift of the carbonyl stretching vibrational modes to lower frequencies in the experimental FT-IR discussed in Section 2.3.

**Figure 3:**
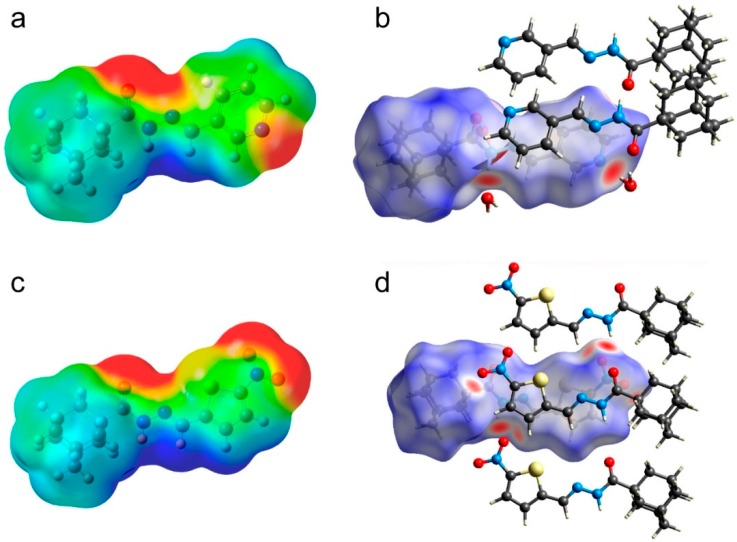
Isodensity (0.0004 e) and Hirshfeld surfaces for compounds **1** (**a** and **b**) and **2** (**c** and **d**). The electrostatic potential is mapped on the isodensity surfaces from red (−0.03 V) to blue (+0.03 V). Atom color code: C (grey), N (blue), O (red), H (white), S (yellow).

Hirshfeld surface analysis was performed in order to semi-quantitatively interpret intermolecular interactions present in the crystal structure. The parameter d_norm_ was mapped throughout the surface, allowing for the recognition of low energy bond hot-spots. The used color code for d_norm_ vs. van der Waal contacts is blue for longer distances, white for equal distances, and red for d_norm_ distances shorter than the sum of the van der Waals radii of the participating atoms. To aid in the recognition of the type of interactions observed in the crystal structure, the electrostatic potential of the gas phase DFT-optimized molecular models was mapped on an isodensity surface (isodensity value = 0.0004 e) with a scale ranging from −0.03 V (red) to +0.03 V (blue). The results are shown in Figure 3.

**Figure 4:**
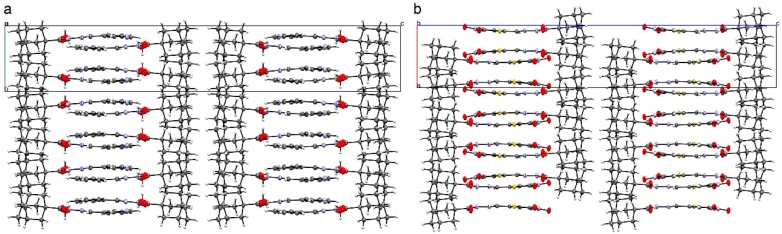
View of crystal packing of compound **1** along the crystallographic a axis (**a**) and compound **2** along the crystallographic b axis (**b**).

The charge is asymmetrically distributed in the compounds. Negative charge is located predominantly around the carbonyl oxygen and pyridine nitrogen atoms for compound **1** and the carbonyl oxygen and nitro-oxygen atoms for **2**, whereas the positive charge surrounds the N-H and C-H bonds in the hydrazine group in both cases. As a consequence, the compounds stack in an antiparallel fashion in the solid state, generating a layered structure along the crystallographic b axis for **1** and the a axis for **2** (Figure 4).

**Figure 5:**
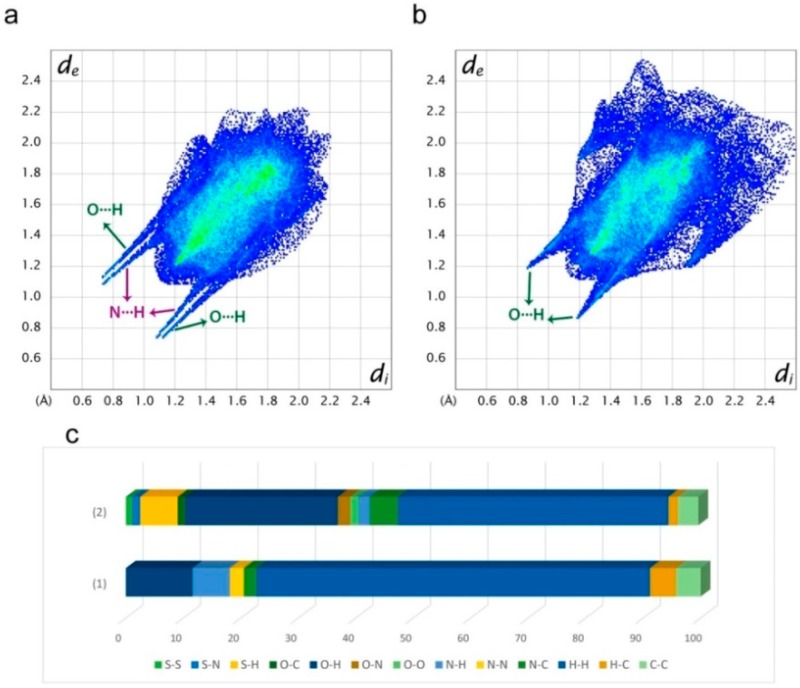
Fingerprint 2D plots for **1** (**a**) and **2** (**b**). Contact percentage contribution of the studied compounds in the surface (**c**).

Fingerprint 2D plots were constructed in order to assess the frequency of the different contacts within the Hirshfeld surface (Figure 5a,b). One of the biggest differences is the splitting of the H bond zone (marked with green and purple arrows) in the **1** plot, where it is possible to discriminate between hydrogen bonds with an O and a N atom acting as H-bond acceptor. This feature is the consequence of the pyridine N atom in the structure of **1**. On the other hand, **2** presents various sulfur-mediated contacts, including S···S, S···N and S···H, as it can be seen on Figure 5c in the contact percentage distribution graph. It is also worth mentioning that there is a significant difference between the contributions of polar contacts in both compounds. For compound **1**, polar contacts account for 18%, while they constitute 42% of the contacts determined for **2**. This is probably the cause of the higher calculated density and symmetry for **2**; **1** crystallizes in a monoclinic space group and **2** in an orthorhombic one.

### 2.3. Infrared Spectra

**Figure 6:**
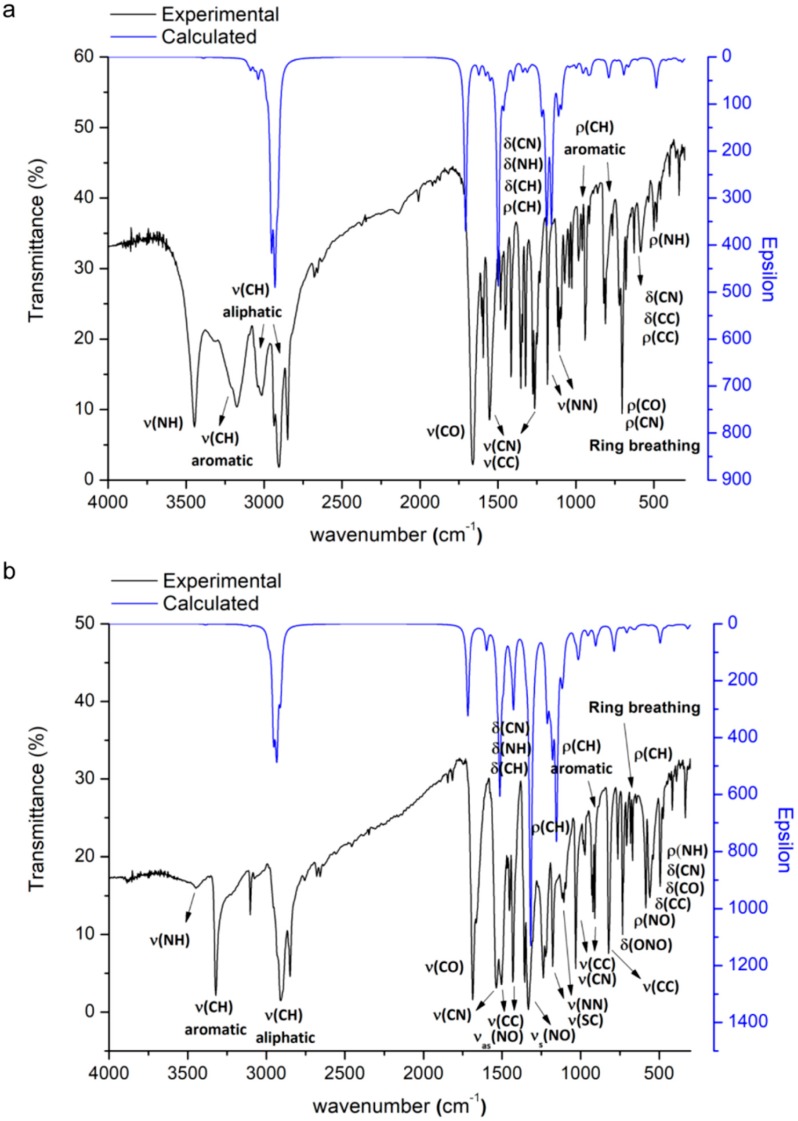
Experimental and calculated IR spectra of compounds **1** (**a**) and **2** (**b**). Theoretical spectra were determined from the gas-phase optimized geometries at the B3LYP/6-31+G(d,p) level. The assignment of the most important IR bands are depicted.

**Figure 7:**
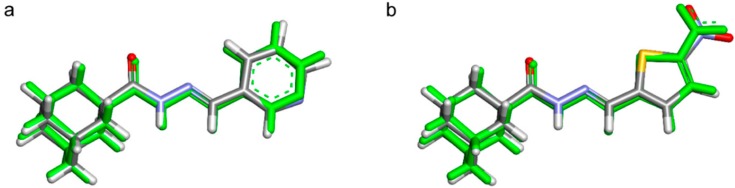
Density functional theory (DFT)-optimized geometries of compounds **1** (**a**) and **2** (**b**) at the B3LYP/6-31+G(d,p) level of theory. The corresponding crystal structures are shown superimposed in green. Atom color code: C (grey), H (white), O (red), N (blue), S (yellow).

Infrared spectroscopy was employed to further structurally characterize both molecules. Such information will also be useful in the future to assess the identity and structure of similar compounds. The infrared spectral profiles of compounds **1** and **2** are depicted in Figure 6. In order to assign the vibrational bands, the spectra were computationally simulated at the B3LYP/6-31+G(d,p) level of theory (blue spectral profiles in Figure 6), and the results were analyzed in depth on the basis of the potential energy distribution (PED; see Tables S1 and S2). Even though the calculations were performed in the gas phase, the computed geometries showed an excellent performance at simulating the solid-state molecular structures of both compounds (see structural discussion below), giving evidence of the validity and robustness of the computational model (Figure 7).

#### 2.3.1. Vibration Modes of the Adamantyl Component

Both IR spectra show bands that indicate the presence of the adamantyl group, related to vibrations involving single C-H and C-C bonds. In detail, the C-H bond stretchings are registered as strong peaks centered at 3015, 2903, and 2849 cm^−1^ for compound **1**, and 2909 and 2849 cm^−1^ for compound **2**. The same vibrational modes have been reported for other adamantyl-containing compounds in the range 2982 to 2902 cm^−1^ [31,32]. Besides, the in-plane C-H bending modes of the adamantyl fragment have been experimentally observed to be scattered over a wide frequency range. In particular, δHCH modes are found between 1508 and 1416 cm^−1^ for compound **1**, and at around 1431 cm^−1^ for compound **2**, in line with previous evidence for similar molecules (reported between 1514 and 1405 cm^−1^) [31,33]. The CH_2_ and C-CH wagging, twisting, and rocking normal vibrations (summarized as torsional and out-of-plane bending modes in Tables S1 and S2) are predicted as several peaks in the ranges 1352 to 862 cm^−1^ (**1**) and 1333 to 910 cm^−1^ (**2**). This is a characteristic spectral feature of the adamantyl fragment, with ρ(C-H) vibrations experimentally located between 1343 and 840 cm^−1^ [31]. Other molecular vibrations that evidence the presence of the adamantyl moiety are the single C-C bond stretching modes. They have been found in the range 1039 to 748 cm^−1^ [31], and are in fact present in the IR spectra of compound **1** (1043–704 cm^−1^) and **2** (1032–669 cm^−1^).

#### 2.3.2. Vibration Modes of the of the *N*′-Methyleneacetohydrazide Moiety

The peaks observed at 3447 cm^−1^ (compound **1**) and 3445 cm^−1^ (compound **2**) can be accounted for by the N-H stretching present in the *N*′-methyleneacetohydrazide moiety. This mode has been computationally predicted at around 3463 cm^−1^, in agreement with the experimental evidence reported for aromatic compounds [34,35]. In-plane HNN bending modes are assigned to the peaks at 1508 cm^−1^ in compound **1** spectrum, and those in the range 1452 to −1431 cm^−1^ in compound **2** spectrum, within the expected frequency interval [36].

Other vibrational modes related to the stretching of N-N and C-N bonds are indicative of the presence of the *N*′-methyleneacetohydrazide group. ν(N-N) normal modes have been recently reported [37] at 1157 and 1012 cm^−1^ and, in fact, they are observed in the spectral profiles of compound **1** (1182 and 1107 cm^−1^) and compound **2** (1179 and 1107 cm^−1^). The sharp and intense peaks found in the spectrum of compound **1** at 1595 and 1263 cm^−1^ can be safely ascribed to ν(C-N) normal modes. The same vibrations are experimentally observed for compound **2** at 1537, 1179, 1032, and 972 cm^−1^. Indeed, the characteristic region for these vibrations has been theoretically predicted between 1646 and 841 cm^−1^ [38].

The presence of the *N*′-methyleneacetohydrazide group in both molecules can also be verified by looking at the intense and sharp peaks at 1663 and 1686 cm^−1^ for compound **1** and compound **2**, respectively. These bands are ascribed to the C=O stretching vibration, in agreement with previous reports on similar systems (ῦ*_exp_* = 1661 cm^−1^) [39]. In general terms, carbonyl compounds show very intense and narrow peaks related to this normal mode, mainly located in the range 1800 to 1600 cm^−1^ [36,39]. The low values of the carbonyl stretching wavenumbers for both molecules can be explained by the conjugation of the C=O bond with the hydrazide moiety, which decreases its double bond character. Another factor that contributes to this effect is the participation of the carbonyl group in the net of H-bonds within the crystal structure (vide infra).

#### 2.3.3. Vibration Modes of the of the Pyridine or 2-Nitrothiophene Groups

The infrared spectrum of compound **1** displays characteristic bands of the pyridyl fragment. The stretching of the single C-H bonds are observed as a broad and intense peak at 3175 cm^−1^, slightly above of the frequency range of the equivalent vibration modes for pyridine (3083–3030 cm^−1^) [37]. The ν(C-N) and ν(C-C) normal modes are also registered at 1555, 1263, 1182, 983, and 941 cm^−1^. Similar vibrations have been reported for pyridine at 1581, 1573, 1030, and 991 cm^−1^ [40]. In-plane bending of C-N-C, C-C-C, and C-C-N bonds of the pyridyl ring were observed at 704 and 584 cm^−1^, near the reported values of 653 and 604 cm^−1^ for pyridine [40].

The infrared bands associated with the 2-nitrothiophene fragment are present in the spectral profile of compound **2**. The C-H stretchings at the thiophene ring appear at 3221 and 3102 cm^−1^, in line with the expected wavenumber range for aromatic compounds [41]. The ν(C=C) normal modes are also observable in the spectrum, mainly near 1504 and 1431 cm^−1^, close to the values already reported for the thiophene ring (1500 and 1430 cm^−1^) [41]. Regarding the C-S stretching mode, the analysis is not straightforward, since it is highly coupled with the C-C and C-N stretching vibrations. However, the peak at 584 cm^−1^ has an important contribution of ν(C-S), and it has been observed between 522 and 680 cm^−1^ for similar systems [33]. Finally, the presence of the nitro group is revealed by the intense bands registered at 1504 and 1452 cm^−1^ (N-O antisymmetric stretching) and at 1333 cm^−1^ (N-O symmetric stretching). Moreover, a sharp and intense peak centered at 733 cm^−1^ is also ascribed to the in-plane O-N-O bending. These wavenumbers are comparable with the ones reported in the literature: ν_asym_(N-O) = 1530 cm^−1^, ν_sym_(N-O) = 1350 cm^−1^, and δ(O-N-O) = 734 cm^−1^ [42].

### 2.4. Electronic Spectra

**Figure 8:**
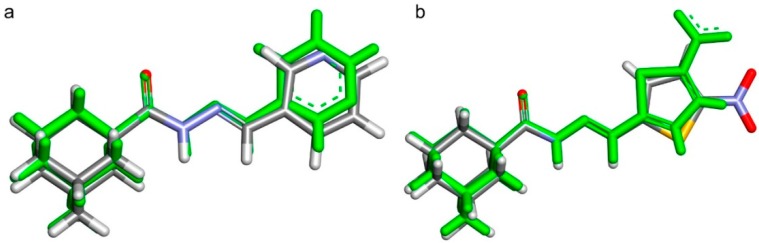
DFT-optimized geometries of **1** (**a**) and **2** (**b**). The corresponding crystal structures are shown superimposed in green. Atom color code: C (grey), H (white), O (red), N (blue), S (yellow).

As a first step to assess the electronic spectra of **1** and **2**, the compounds’ geometries were DFT-optimized in methanolic medium. The results indicate that, in solution, the pyridyl and 2-nitrothiophene rings rotate 180° as a consequence of the absence of the intermolecular interactions observed in the solid state (Figure 8).

**Figure 9:**
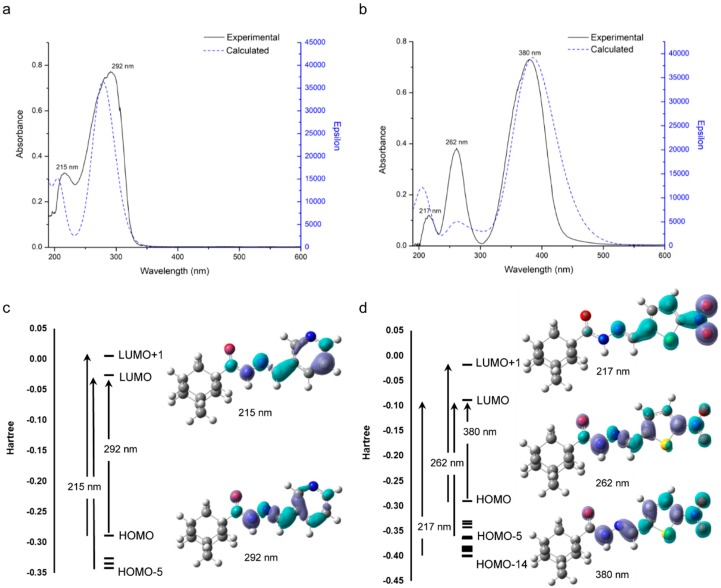
UV-vis spectra of a 10 μg/mL methanolic solution of **1** (**a**) and **2** (**b**). Experimental and calculated spectra (CAM-B3LYP-SMD/6-311+G(d,p)) are depicted as solid and dashed lines, respectively. For the theoretical spectra, the half-width at half-height is 0.333 eV. In (**c**) and (**d**), the molecular orbitals involved in the different electronic transitions and their energies are shown for both systems. The electron density difference maps (EDDM) associated with the electronic transitions are also depicted. The electron density changes from purple to cyan upon light absorption. Atom color code: C (grey), H (white), O (red), N (blue), S (yellow).

UV-vis absorption spectra of compounds **1** and **2** in methanol were recorded in the 200–600 nm range. The results are shown in Figure 9a,b. To aid in the discussion, a holistic view of the light absorption process was furnished by single-point TD-DFT calculations carried out in solution. That allowed us to compute the electron density difference maps (EDDM) associated with the most probable electronic transitions, which show, in a picturesque way, how the electron density changes during the light absorption (it moves from purple to cyan zones; see Figure 8a,b). As expected, the saturated adamantyl group does not contribute to the registered UV-vis absorption bands [33].

The spectral profile of compound **1** shows two main bands centered at λ_max_ = 215 and λ_max_ = 292 nm, which are satisfactorily simulated by the computational model (Figure 9a). The first one (λ_calculated_ = 206 nm) is predicted to involve π → π* electronic transitions, with two main associated electronic promotions: HOMO-5 → LUMO (66% contribution) and HOMO → LUMO+1 (17% contribution). On the other hand, the experimental absorption band at 292 nm is computationally predicted at 279 nm, and it comprises one electronic transition brought about by a π → π* HOMO-LUMO electronic promotion (96% contribution). According to the EDDM surfaces of both UV-visible bands, during the light absorption, the electron density flows within the π-delocalized system, from the *N*′-methyleneacetohydrazide moiety to the pyridyl ring at 292 nm and backwards at 215 nm.

The electronic spectra of compound **2** exhibits three absorption bands, showing a satisfactory fit with the calculated spectral profile (Figure. 8b). The most energetic one was registered at 217 nm and computationally predicted at 213 nm. It is ascribed to a π → π* electronic transition that has a 92% contribution of the electron promotion from HOMO-14 to LUMO. Interestingly, the EDDM surface reveals that, during the light absorption associated with this band, the electron density is transferred from the nitro group towards the thiophene ring and the methyleneacetohydrazide moiety. The band located around 262 nm (λ_calculated_ = 261 nm) is brought about by the contribution of two electronic π → π* promotions: HOMO-5 → LUMO (23% contribution) and HOMO → LUMO+1 (72% contribution), similarly to the band registered at 215 nm for compound **1**. Finally, the third band is centered at 380 nm (λ_calculated_ = 384 nm) and exhibits a tail that extends toward the visible range. The associated light absorption mechanism is similar to the one described for the 292 nm absorption band of compound **1**, and entails a HOMO-to-LUMO electronic transition (93% contribution). It is worth noting that, according to the calculated EDDM surfaces, there is a retrodonation of electron density from the methyleneacetohydrazide and thiophene moieties to the nitro group upon light absorption at λ = 262 and 380 nm.

### 2.5. In Vitro Antimicrobial and Antifungal Activity

The in vitro antimicrobial activity of compounds **1** and **2** was assessed against the standard Gram-positive bacterial strains of the American type culture collection (ATCC), *Staphylococcus aureus* ATCC 6571, *Bacillus subtilis* ATCC 5256, and *Micrococcus luteus* ATCC 27141, the Gram-negative bacterial strains, *Escherichia coli* ATCC 8726 and *Pseudomonas aeruginosa* ATCC 27853, and the yeast-like pathogenic fungus *Candida albicans* MTCC 227. The primary screening was performed using the semi-quantitative agar-disc diffusion method with Müller–Hinton agar medium [43]. The results of the preliminary antimicrobial screening of compounds **1**, **2** (200 μg/disc), the antibacterial antibiotics gentamicin sulfate and ampicillin trihydrate, and the antifungal drug clotrimazole (100 μg/disc) are outlined in Table 3.

The main features of the results of the antimicrobial activity testing show that compounds **1** and **2** exhibit potent activity against the tested Gram-positive bacteria, with growth inhibition zones and minimal inhibitory concentration (MIC) values comparable to the positive control antibiotics. Compound **2** specifically displays broad-spectrum antibacterial activity. The inhibitory activity of the compounds against yeast-like pathogenic fungus *C. albicans* was lower than their antibacterial activity, with moderate and weak activities for **1** and **2,** respectively. Taking into consideration the lipophilicity of both compounds (calculated log *P* values: 2.513 (**1**) and 3.3405 (**2**)), it could be concluded that the inhibitory activity against the Gram-negative bacteria is correlated to their lipophilicty, the opposite relationship is observed for the antifungal activity. The values of the minimal inhibitory concentration (MIC) in Müller–Hinton broth [44] for the tested compounds (Table 4) were correlated to the results obtained in the preliminary screening.

## 3. Materials and Methods

### 3.1. Instrumentation

Melting points (°C, uncorrected) were measured in open glass capillaries using a Branstead 9100 Electrothermal melting point apparatus. Nuclear magnetic resonance (NMR) spectra were obtained on a Bruker Ascend 700 NMR spectrometer at 700.17 MHz for ^1^H and 176.08 MHz for ^13^C, using DMSO-*d_6_* as solvent. Monitoring the reactions and checking the purity of the final products were carried out by thin layer chromatography (TLC) using silica gel precoated aluminum sheets (60 F_254_, Merck) and visualization with ultraviolet light (UV) at 365 and 254 nm. Single-crystal X-ray diffra ction data were collected on a Rigaku OD XtaLAB Synergy, Dualflex, Pilatus 200K diffractometer, using a single wavelength X-ray source (Cu Kα radiation: λ = 1.54184 Å) from a micro-focus sealed X-ray tube and an Oxford liquid-nitrogen Cryostream cooler. Fourier transform infrared (FT-IR) spectra were measured using KBr pellets in the 400–4000 cm^−1^ region on a Shimadzu IR Prestige 21 spectrometer. UV-Vis spectra were determined using a Shimadzu UV-1601 PC UV-Visible double-beam spectrophotometer with matched 1 cm path-length quartz cell. The experimental details of the determination of in vitro antimicrobial activity are given in Supplementary Materials.

### 3.2. Synthesis, Crystallization, and Single Crystal X Ray Determination

#### 3.2.1. *E*-*N*′-[(Pyridine-3-yl)methylidene]adamantane-1-carbohydrazide **1**

Pyridine-3-carboxaldehyde (1.07 g, 0.01 mol) was added to a solution of adamantane-1-carbohydrazide (1.94 g, 0.01 mol), in ethanol (15 mL), and the mixture was heated under reflux for two hours. The mixture was then concentrated to half the original volume and water (5 mL) was added and allowed to stand overnight. The precipitated crude product was filtered, washed with water, dried, and crystallized from EtOH/H_2_O to yield (2.32 g, 82%) of the monohydrate of compound **1** as transparent colorless needle crystals. The anhydrous compound was obtained after drying in a desiccator for 24 h. Mp: 185–187 °C. ^1^H-NMR: δ 11.01 (s, 1H, NH), 8.81 (s, 1H, CH=N), 8.61 (s, 1H, Pyridine-H), 8.47 (d, 1H, Pyridine-H, *J* = 4.5 Hz), 8.01 (s, 1H, Pyridine-H), 7.48 (t, 1H, Pyridine-H, *J* = 4.5 Hz), 2.03 (s, 3H, Adamantane-H), 1.88 (s, 6H, adamantane-H), 1.72 (s, 6H, adamantane-H). ^13^C-NMR: δ 173.86 (C=O), 150.95, 149.05, 133.73, 130.79, 124.48 (Pyridine-C), 144.35 (CH=N), 39.77, 38.73, 36.53, 28.05 (adamantane-C).

#### 3.2.2. *E*-*N*′-[(5-Nitrothiophen-2-yl)methylidene]adamantane-1-carbohydrazide **2**

5-Nitrothiophene-2-carboxaldehyde (1.57 g, 0.01 mol) was added to a solution of adamantane-1-carbohydrazide (1.94 g, 0.01 mol), in ethanol (15 mL), and the mixture was heated under reflux for one hour. On cooling, the precipitated crude product was filtered, washed with cold ethanol, dried, and crystallized from EtOH to yield (3.17 g, 95%) of compound **2** as transparent pale yellow crystals. Single crystals suitable for X-ray diffraction were obtained by slow evaporation of a solution of the title compound in EtOH/CHCl_3_ (1:2, *v/v*) at room temperature. Mp: 233–235 °C. ^1^H-NMR: δ 11.24 (s, 1H, NH), 8.68 (s, 1H, CH=N), 8.12 (d, 1H, Thiophene-H, *J* = 4.2 Hz), 7.51 (d, 1H, Thiophene-H, *J* = 4.2 Hz), 2.02 (s, 3H, Adamantane-H), 1.87 (s, 6H, adamantane-H), 1.68–1.73 (m, 6H, adamantane-H). ^13^C-NMR: δ 174.18 (C=O), 151.03, 147.73, 130.99, 129.58 (Thiophene-C), 140.63 (CH=N), 39.74, 38.55, 36.42, 27.96 (adamantane-C).

The selected suitable single crystals of compounds **1** and **2** were mounted using polybutene oil on a flexible loop fixed on a goniometer head and immediately transferred to the diffractometer. Pre-experiment, data collection, data reduction, and analytical absorption correction [45] were performed with the program suite CrysAlisPro [46] using the Olex2 program [47]. The structures were solved with the SHELXT [48] small molecule structure solution program and refined with the SHELXL 2018/3 program package [49] by full-matrix least-squares minimization on F^2^. *PLATON* [50] was used to check the result of the X-ray analysis. For more details about the data collection and refinement parameters, see the CIF files.

### 3.3. Computational Details

#### 3.3.1. Hirshfeld Surface Analysis

Hirshfeld surfaces were calculated in order to provide a semi-quantitative analysis of intermolecular interactions using CRYSTAL EXPLORER17 [51]. The normalized contact distance (*d*_norm_), mapped throughout the surface, is defined as:

$$d_{\mathrm{norm}}=\frac{d_{i}-r_{i}^{\mathrm{vdW}}}{r_{i}^{\mathrm{vdW}}}+\frac{d_{e}-r_{e}^{\mathrm{vdW}}}{r_{e}^{\mathrm{vdW}}}$$

where d_e_ and d_i_ represent the distances from a point on the surface to the nearest nucleus outside and inside the surface, respectively, and r^vdW^ corresponds to the van der Waals (vdW) radii of the atoms involved. Based on that information, a 2D fingerprint plot of d_e_ vs. d_i_ distances within the surface and their frequency was determined to provide quantitative information on the interactions throughout the crystal structure [52,53].

#### 3.3.2. DFT Calculations

Starting geometries for compounds **1** and **2** were extracted from the X-ray crystallographic data and fully optimized without symmetry constraints by means of the density functional theory method (DFT) [54], employing the B3LYP functional [55] and the 6-31+G** basis set [56]. The calculations were performed in the gas phase, with an ultrafine integration grid, employing the Gaussian 09 code [57]. To make a detailed assignation of the infrared spectra, the calculated vibrational modes were characterized by means of the potential energy distribution (PED) with the program VEDA 4 [58]. The computed vibrational frequencies were scaled using a factor of 0.964 [59].

To model the electronic spectra in methanol, the X-ray crystallographic structures of both molecules were pre-optimized in solution by molecular mechanics, employing the MMFF94X forcefield (energy gradient = 0.01 kcal/mol/Å^3^), as implemented in MOE [60]. The solvent was modeled by means of the Generalized Born model (ε = 33.1). To explore the conformational space, a search on the potential energy surface was carried out with the LowModeMD method [61] using the same forcefield and solvation method, without cut-offs, and with a RMS gradient = 0.005 kcal/mol/Å^3^ (rejection limit = 100; RMSD limit = 0.25 Å; energy window = 7 kcal/mol; iteration limit = 10,000). The number of conformations found were 4 for **1** and 16 for **2**. Then, the most stable conformers were re-optimized in solution by DFT (B3LYP/6-31+G**). The influence of the solvent was simulated by an IEFPCM method (implicit solvation), with radii and non-electrostatic terms from Truhlar and coworkers’ SMD solvation model [62]. Finally, single-point TD-DFT calculations were carried out on the optimum geometries to simulate the UV-visible light absorption process [55]. Fifty singlet-to-singlet electronic transitions were considered and their vertical energies were computed in methanol at the CAM-B3LYP-SMD/6-311+G(d,p) level of theory [63]. An in-depth analysis of the light absorption mechanism was carried out with the aid of the GaussSum 3.0 program [64]. The electron density difference maps (EDDM) and the molecular orbitals related to the most probable electronic transitions were computed, considering the contribution of three molecular fragments, namely: i) pyridyl (**1**) or 2-nitrothiophen-2-yl (**2**) group, ii) *N*′-methyleneacetohydrazide moiety, and iii) adamantyl group. In all cases, the nature of the stationary states was verified by analytically computing the Hessian matrix. The optimized geometries were found to have only real vibrational frequencies. The computational results were rendered with Discovery Studio Visualizer and Gaussview 5.0 [65].

## 4. Conclusions

In the present study, two novel *N*′-[(heteroaryl)methylene]adamantane-1-carbohydrazides (compounds **1** and **2**) were prepared and tested for growth inhibitory activity. Both molecules displayed potent antibacterial activity against a panel of pathogenic Gram-positive and Gram-negative bacteria and the yeast-like pathogenic fungus *Candida albicans*. Compound **2** showed potent broad-spectrum antibacterial activity and marginal antifungal activity, while compound **1** displayed potent activity against the tested Gram-positive bacterial strains and moderate activity against the tested Gram-negative bacteria and *Candida albicans*. According to the preliminary antimicrobial results, the tested compounds are considered to be good candidates as new antibacterial agents.

Compounds **1** and **2** were fully characterized by applying a wide range of experimental and computational methods. They revealed that the electron density is asymmetrically distributed in both molecules, giving rise to positively and negatively charged zones that interact through polar contacts in the crystalline state. This interaction scheme extends along the lattice, compelling the molecules to stack themselves in an antiparallel fashion. Nevertheless, there is a differential contribution of the polar contacts to the stability of the lattice: **1** (18%) and **2** (42%), modulating the density and symmetry observed in the crystals. Additionally, both molecules act as chromophores, showing intense UV-vis light absorption bands brought about by electronic transitions within the π-delocalized system.

Further investigations, including the preparation of novel related derivatives, are required for optimization of the antibacterial activity and for exploration of the mechanism of the compounds’ biological activity. We are currently working on those lines, and the results will be published in due course.

## Figures and Tables

**Table 1:** Crystal data and structure refinement parameters of compounds **1** and **2**.

Structural Parameters	Compound 1	Compound 2
Empirical formula	C_17_H_23_N_3_O_2_	C_16_H_19_N_3_O_3_S
Formula weight	301.38	333.40
Temperature	160(1) K	160 (1) K
Wavelength	1.54184 Å	1.54184 Å
Crystal system	Monoclinic	Orthorhombic
Space group	I 1 2/a 1	Pbca
Unit cell dimensions	*a* = 11.34690(10) Å *b* = 6.66810(10) Å *c* = 40.4828(5) Å *β* = 94.9240(10)°	*a* = 6.78143(15) Å *b* = 11.4079(3) Å *c* = 39.1760(8) Å *β* = 90°
Volume (Å^3^)	3051.72(7)	3030.74(12)
*Z*	8	8
Calculated density (mg/m^3^)	1.312	1.461
Absorption coefficient (mm^−1^)	0.700	2.073
*F*(000)	1296	1408
Crystal size (mm^3^)	0.13 × 0.04 × 0.02	0.12 × 0.065 × 0.015
Theta range for data collection	4.385 to 74.475°	4.515 to 74.488°
Index ranges	−14 ≤ h ≤ 14, −8 ≤ k ≤ 7, −50 ≤ l ≤ 49	−8 ≤ h ≤ 7, −14 ≤ k ≤ 14, −47 ≤ l ≤ 48
Reflections collected	25245	13432
Independent reflections	3122 [*R*(int) = 0.0342]	3102 [*R*(int) = 0.0384]
Completeness	100.0%	100.0%
Max. and min. transmission	0.985 and 0.929	0.973 and 0.846
Data/restraints/parameters	3122/0/211	3102/0/212
Goodness-of-fit on *F*^2^	1.045	1.071
Final *R* indices [*I > 2σ(*I*)*]	*R*_1_ = 0.0365, w*R*_2_ = 0.0957	*R*_1_ = 0.0395, w*R*_2_ = 0.1001
*R* indices (all data)	*R*_1_ = 0.0424, w*R*_2_ = 0.0993	*R*_1_ = 0.0493, w*R*_2_ = 0.1046
Largest diff. peak and hole (e.Å^−3^)	0.252 and −0.216	0.505 and −0.273
CCDC number	1992005	1992009

**Table 2:** Hydrogen bonding geometrical parameters for compounds **1** and **2** (Å, °).

D-H···A	*d*(D-H)	*d*(H···A)	*d*(D···A)	<(DH···A)
Compound **1**				
N(3)-H(3A) **···**O(2)#1^a^	0.881(17)	1.959(17)	2.8345(14)	172.8(15)
O(2)-H(2A) **···**N(1)#2^a^	0.90(2)	1.95(2)	2.8368(14)	165(2)
O(2)-H(2B) **···**O(1)	0.89(3)	1.93(3)	2.8118(16)	172(2)
Compound **2**				
N(3)-H(3) **···**O(2)#1^b^	0.82(3)	2.19(3)	2.881(2)	142(3)

Symmetry transformations used to generate equivalent atoms: **1**: #1^a^ x − 1/2, −y + 1, z, #2^a^ –x + 1, y + 1/2, −z + 1/2; **2**: #1^b^ –x + 1, y − 1/2, −z + 3/2.

**Table 3:** In vitro diameter of growth inhibition zones of compounds **1**, **2** (200 μg/8 mm disc), the broad-spectrum antibacterial drugs Gentamicin sulfate and Ampicillin trihydrate, and the antifungal drug Clotrimazole (100 μg/8 mm disc) against *Staphylococcus aureus* ATCC 6571 (*SA*), *Bacillus subtilis* ATCC 5256 (*BS*), *Micrococcus luteus* ATCC 27141 (*ML*), *Escherichia coli* ATCC 8726 (*EC*), *Pseudomonas aeruginosa* ATCC 27853 (*PA*), and the yeast-like pathogenic fungus *Candida albicans* MTCC 227 (*CA*).

	Diameter of Growth Inhibition Zone (mm)					
	*SA*	*BS*	*ML*	*EC*	*PA*	*CA*
Compound **1**	28	26	23	12	14	15
Compound **2**	31	32	22	22	19	11
Gentamicin sulfate	27	26	20	22	21	NT
Ampicillin trihydrate	22	23	20	16	16	NT
Clotrimazole	NT	NT	NT	NT	NT	21

NT: not tested.

**Table 4:** The minimal inhibitory concentration (MIC) of compounds **1**, **2**, the broad-spectrum antibacterial drugs Gentamicin and Ampicillin, and the antifungal drug Clotrimazole against *Staphylococcus aureus* ATCC 6571 (*SA*), *Bacillus subtilis* ATCC 5256 (*BS*), *Micrococcus luteus* ATCC 27141 (*ML*), *Escherichia coli* ATCC 8726 (*EC*), *Pseudomonas aeruginosa* ATCC 27853 (*PA*), and the yeast-like pathogenic fungus *Candida albicans* MTCC 227 (*CA*).

	MIC, µg/mL (µM)					
	*SA*	*BS*	*ML*	*EC*	*PA*	*CA*
Compound **1**	1 (3.53)	1 (3.53)	2 (7.06)	64 (225.85)	>64	64 (225.85)
Compound **2**	0.5 (1.50)	0.5 (1.50)	1 (3.0)	2 (6.0)	2 (6.0)	>64
Gentamicin	1 (2.09) ^1^	2 (4.19) ^1^	2 (4.19) ^1^	0.5 (1.05) ^1^	0.5 (1.05) ^1^	NT
Ampicillin	2 (5.72) ^2^	1 (2.86) ^2^	2 (5.72) ^2^	8 (22.90) ^2^	8 (22.90) ^2^	NT
Clotrimazole	NT	NT	NT	NT	NT	4 (11.60)

^1^ μM values were calculated based on Gentamicin base, ^2^ μM values were calculated based on anhydrous Ampicillin, NT: not tested.

## Supplementary Materials

The experimental details of the determination of in vitro antimicrobial activity, ^1^H NMR, ^13^C-NMR spectra and the infrared spectroscopy data of compounds **1** (Table S1) and **2** (Table S2) are available online. The supplementary crystallographic data for compounds **1** (CCDC 1992005) and **2** (CCDC 1992009) can be obtained free of charge from The Cambridge Crystallographic Data Centre at: www.ccdc.cam.ac.uk.
