# Supplementary material for: Synthesis and Structure Insights of Two Novel Broad-Spectrum Antibacterial Candidates Based on (E)-N′-[(Heteroaryl)methylene]adamantane-1-carbohydrazides
Source: Molecules. 2020 Apr 22;25(8):1934. doi: 10.3390/molecules25081934 (PMC7221572; doi:10.3390/molecules25081934)
Supplement: Supplementary file 1 [file molecules-25-01934-s001.pdf]

Supplementary Materials

Synthesis and Structure Insights of Two Novel Broad-Spectrum Antibacterial Candidates Based on (*E*)-*N*'-[(Heteroaryl)methylene]adamantane-1-carbohydrazides

Table S1. Experimental and calculated infrared wavenumbers of compounds **1**.

**Infrared wavenumbers (cm⁻¹)**		**Assignment (%PED)**[a]
Experimental	Calculated	
3447	3387	νNH(100)
3175	3101	νCH(89)sym in py
	3086	νCH(84)asym in py
	3060	νCH(91)asym in py
	3038	νCH(99) in py
3015<br>2903<br>2849	2985	νCH(98)asym in Ad
	2955	νCH(90)asym in Ad
	2952	νCH(94)asym in Ad
	2948	νCH(92)sym/asym in Ad
	2946	νCH(90)asym in Ad
	2935	νCH(89)sym in Ad
	2930	νCH(92)sym in Ad
	2920	νCH(99) in NN
	2910	νCH(96)sym in Ad
	2909	νCH(92)sym in Ad
	2905	νCH(90)sym in Ad
	2904	νCH(85)asym in Ad
1663	1707	νCO(88) in NN
1595	1622	νCN(73) in NN
1555	1579	νCN + νCC (62) in py
	1551	νCN + νCC (63) in py
1508<br>1483<br>1452<br>1416	1498	δHNC + δHNN + δCNN (68) in NN
	1466	δHCH(87) in Ad
	1462	δHCC + δHCN (44) in py
	1445	δHCH(88) in Ad
	1443	δHCH(90) in Ad
	1431	δHCH(89) in Ad
	1430	δHCH(89) in Ad
	1401	δHCC + δHCN (41) in py
1352<br>1323	1348	δHCC(62) in Ad
	1343	τHCCC + γCCCH (36) in Ad
	1340	δHCN(46) in NN
	1330	τHCCC(59) in Ad
	1327	δHCC(78) in Ad
	1312	δHCC + δHCN (76) in py and NN
	1302	δHCC(92) in Ad
	1298	δHCC(67) in Ad
	1295	δHCC(27) in Ad
	1270	δHCC(60) in Ad

	1266	νCN + νCC (83) in py + δHCC(54) in Ad
1263	1245	δHCC(42) in Ad
	1233	δHCC(47) in Ad
	1219	νCN + νCC+ νNN (35) in py and NN
1182	1188	νCC+ νNN (21) in py and NN
	1180	δHCC + δHCN (39) in py
1107	1163	δHCC(66) in Ad
	1112	νNN(43) in NN
	1099	δHCC(86) in Ad
	1093	νCN + νCC+ νNN (31) in py and NN
	1090	δHCC(91) in Ad
	1085	γCCCH (25) in Ad
1072	1079	τHCCC + γCCCH (21) in Ad
1043	1074	γCCCH (31) in Ad
1026	1041	νCN + νCC+ νNN (31) in py and Ad
983	1025	νCN + νCC (71) in py
959	1013	νCN + νCC (51) in Ad and NN
	1007	νCC(55) in Ad
	998	δCCC + δCNC (77) in py
	975	τHCCH + τHCCC + τHCNC (83) in py
	956	γCCCC(29) in Ad
	949	νCN + νCC (38) in Ad and NN
	948	νCC(38) in Ad
	945	τHCCH + τHCCC + τHCNC (79) in py
941	923	τHCCH + τHCCC + τHCNC + τHCNN (79) in py and NN
	909	νCC(59) in Ad
	907	νCC(59) in Ad
	898	τHCCH + τHCCC + τHCNC + τHCNN (85) in py and NN
	864	τCCCH (75) in Ad
862	861	τCCCH (70) in Ad
	860	τCCCH (65) in Ad
	839	νCC(47)
	793	νCC(76) in Ad
810	791	τHCCC + γCCCH (75) in py
	790	νCC(63)
	785	νCC(62)
	738	νCC(87) in Ad (ring breathing)
	700	γOCNC(57) in Ad
704	692	τCCNC + τCNCC + τCCCN (85) in py
	663	δCCN + δCCC + δCNN (74)
	656	δCCN + δNCO + δCCC (51)
626	631	τCCCC + γCCCC (53) in Ad
584	629	δCCC(62) in Ad
	605	δCNC + δCCC (83) in py
500	505	τCCNC + τCCNN + τCCCC (83) in py
482	484	τHNCC + τHNNC (92) in NN

[1] The assignment was made starting from the computational results at B3LYP/6-31+G** level of theory. Only those modes with %PED ≥ 20% were taken into account. ν: stretching; γ: out-of-plane bending; δ: in-plane bending; τ: torsion; sym: symmetric; asym: antisymmetric. py: pyridyl; NN: N'-methyleneacetohydrazide; Ad: adamantyl.

Table S2. Experimental and calculated infrared wavenumbers of compounds **2**.

**Infrared wavenumbers (cm⁻¹)**		**Assignment (%PED)[a]**
Experimental	Calculated	
3445	3387	νNH(100)
3221	3142	νCH(97) in 2NT
3102	3105	νCH(97) in 2NT
2909	2986	νCH(96)asym in Ad
	2956	νCH(88)asym in Ad
	2954	νCH(88)asym in Ad
	2953	νCH(87)asym in Ad
	2949	νCH(94)asym in Ad
	2948	νCH(92)sym/asym in Ad
	2947	νCH(90)asym in Ad
	2939	νCH(99) in NN
	2936	νCH(86)sym in Ad
	2932	νCH(86)sym in Ad
	2931	νCH(91) in Ad
2849	2912	νCH(93)sym in Ad
	2909	νCH(89)sym in Ad
	2905	νCH(86)sym in Ad
	2903	νCH(86)sym in Ad
1686	1718	νCO(88) in NN
1537	1598	νCN(76) in NN
1504	1530	νNO asym + νCC (68) in 2NT
1452	1513	νNO asym + νCC (57) in 2NT + δHNC + δHNN + δCNN (20) in NN
1431	1491	νNO asym + νCC (25) in 2NT + δHNC + δHNN + δCNN (40) in NN
	1466	δHCH(91) in Ad
	1446	δHCH(86) in Ad
	1443	δHCH(89) in Ad
	1431	δHCH(73) in Ad
	1430	δHCH(81) in Ad
	1427	νCC(47) in 2NT
1333	1350	δHCC(28)
	1348	δHCC(47) in Ad
	1347	δHCC(27)
	1342	δHCC(69) in Ad
	1329	τHCCC(63) in Ad
	1327	δHCC(73) in Ad
	1318	νNOsym(71) in 2NT
1238	1302	δHCC(66) in Ad
	1298	δHCC(68) in Ad
	1296	δHCC(52) in Ad
	1286	δHCN(45) in NN
	1270	δHCC(55) in Ad
	1269	δHCC(52) in Ad
	1266	δHCC(50) in Ad
	1244	δHCC(50) in Ad
	1233	τHCCH + τHCCC (43) in Ad
1179	1213	νCC + νSC (59) in 2NT
	1196	δHCC + δHCN (62) in 2NT and NN

	1180	νCC + νCN+ νNN (26) in NN
	1163	δHCC(57) in Ad
	1154	νCC + νCN+ νNN (20) in NN
	1118	νNN(52) in NN
	1110	νCN + νSC+ νNO (58) in 2NT
	1099	δHCC(75) in Ad
1107	1091	δHCC(79) in Ad
	1085	δHCC(55) in Ad
	1079	δHCC(52) in Ad
	1075	δHCC(55) in Ad
	1036	δCCC(22)
	1018	δHCC(39) in 2NT
1032	1012	νCC(61) in Ad
	1009	νCN + νCC (43) in Ad and NN
	1006	νCC(53) in Ad
	955	νCC(33) + δCCC(22) in Ad
972	948	νCC(33) in Ad
	947	νCN + νCC (32) in Ad and NN
	910	νCN + νCC (45) in Ad and NN
	909	νCC(62) in Ad
	905	νCC(55) in Ad
910	902	τHCNN + τHCCC + τHCCN (88) in 2NT
	886	τHCCH + τHCCN + τHCNN (90) in 2NT
	864	δHCC + δCCC (51) in Ad
	861	τHCCH + τHCCC + γCCCH (51) in Ad
	860	τHCCC + τCCCC + γCCCH (47) in Ad
824	799	νCC(26) in Ad
	792	νCC(73) in Ad
733	789	δONO(58) in 2NT
707	787	τHCCN + τHCCC (92) in 2NT
	785	νCC(71) in Ad
669	747	δCCC(55) in 2NT
	738	νCC(79) in Ad (ring breathing)
	707	γCNO + γONO (90) in 2NT
	698	τHNCO + τCCCC + τHNCC (54) in Ad and NN
	673	νSC(43) in 2NT
584	664	νSC + νCC (41) in 2NT
	651	νCC(42) + δCCC + δNCO (24)
	631	τCCCC (54) in Ad
	629	δCCC(63) in Ad
559	570	τHCCC + τHCCS + τCCCC + τCCNN (92) in NN and 2NT
494	524	δCCC + δCNO + δCCN + δNCO (53) in NN and 2NT
	494	τHNNC + τHNCO + τCCNN (68)

[1] The assignment was made starting from the computational results at B3LYP/6-31+G** level of theory. Only those modes with %PED ≥ 20% were taken into account. ν: stretching; γ: out-of-plane bending; δ: in-plane bending; τ: torsion; sym: symmetric; asym: antisymmetric. 2NT: 2-nitrothiophene; NN: N'-methyleneacetohydrazide; Ad: adamantyl.

^{1}H NMR Spectra of compound **1** in DMSO-d6.

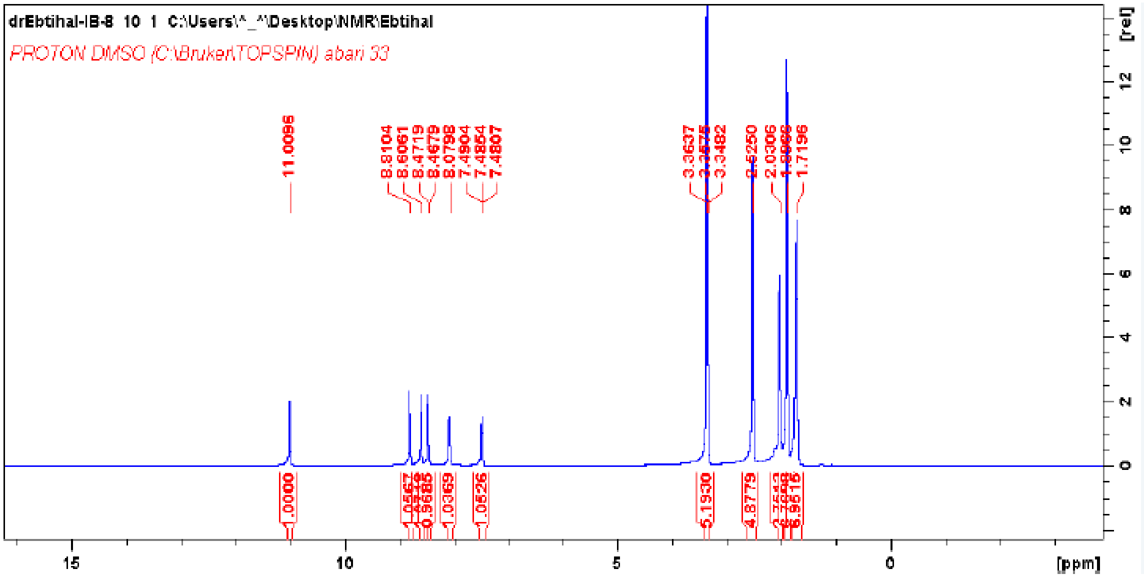

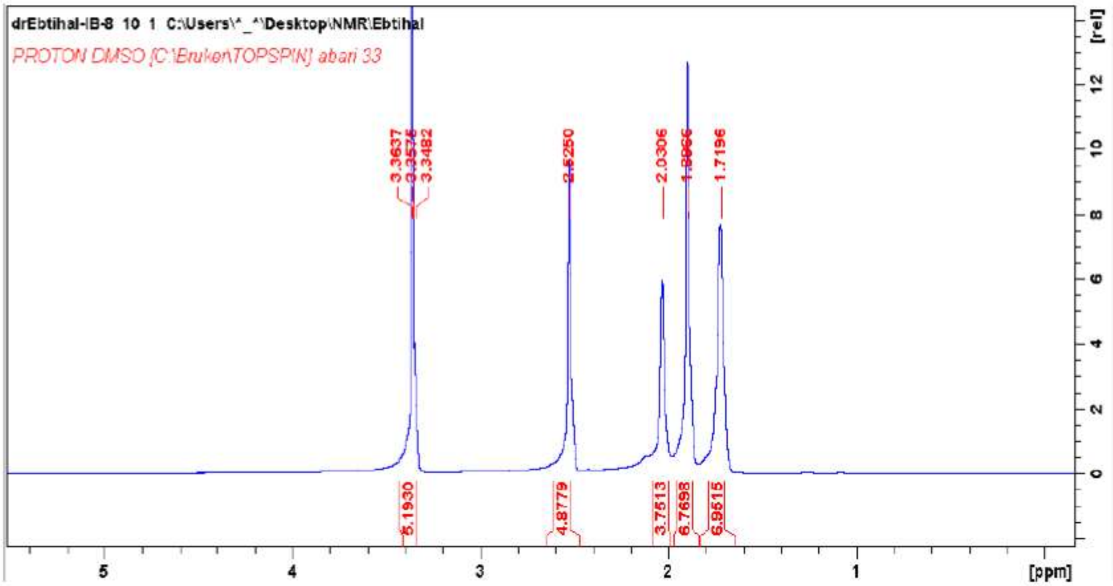

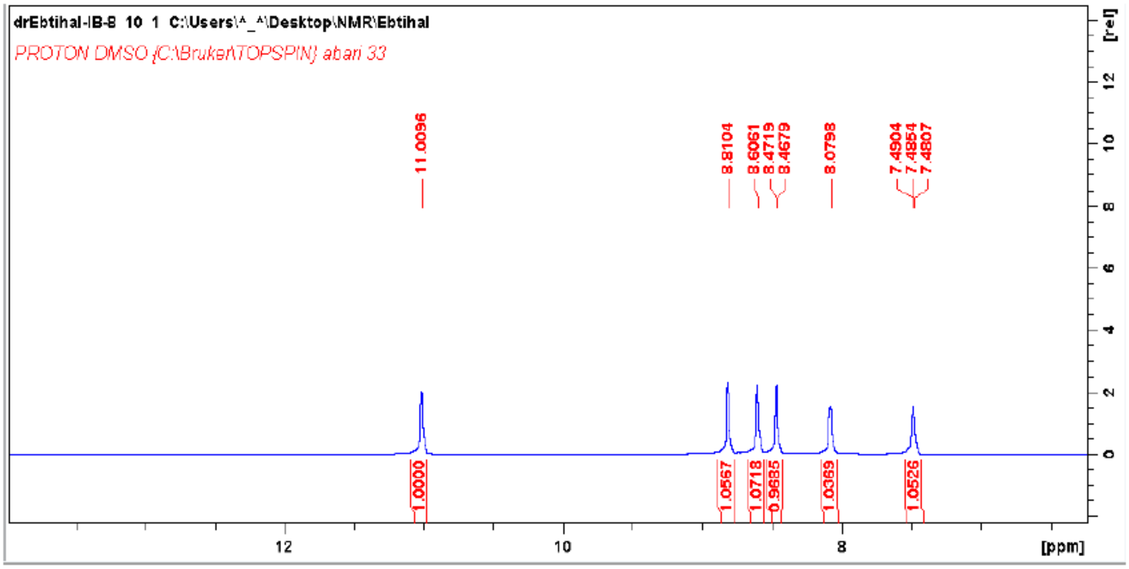

^{13}C NMR Spectra of compound **1** in DMSO-d_6.

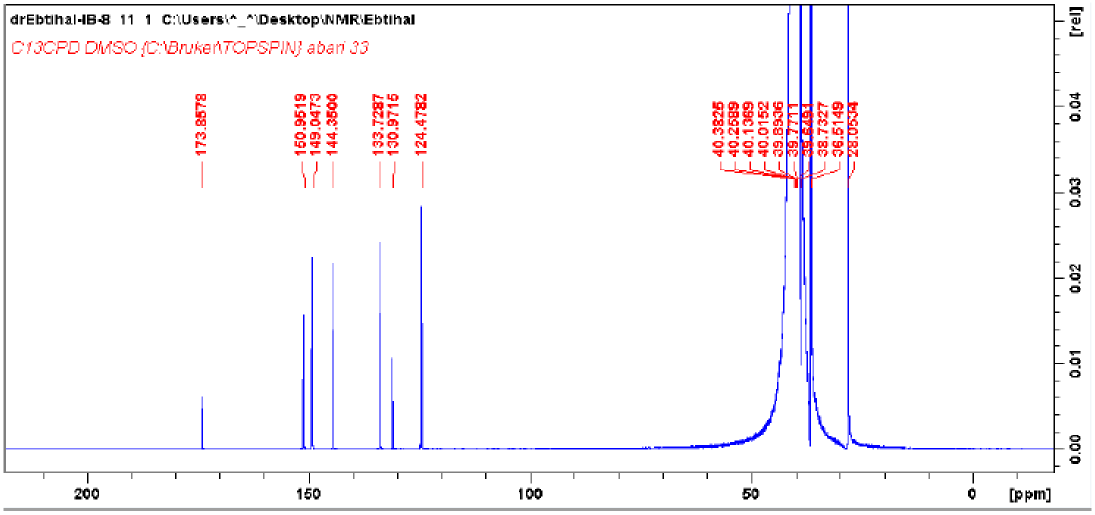

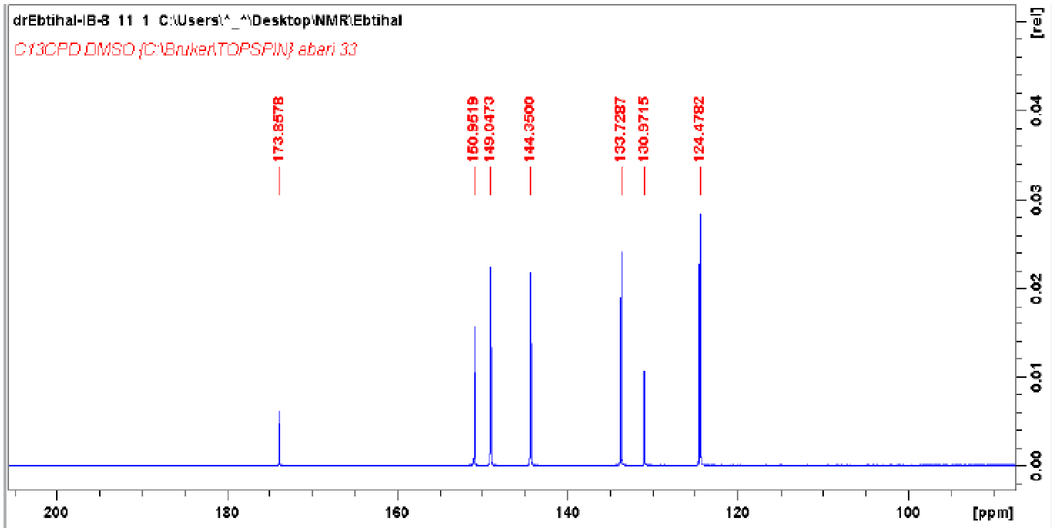

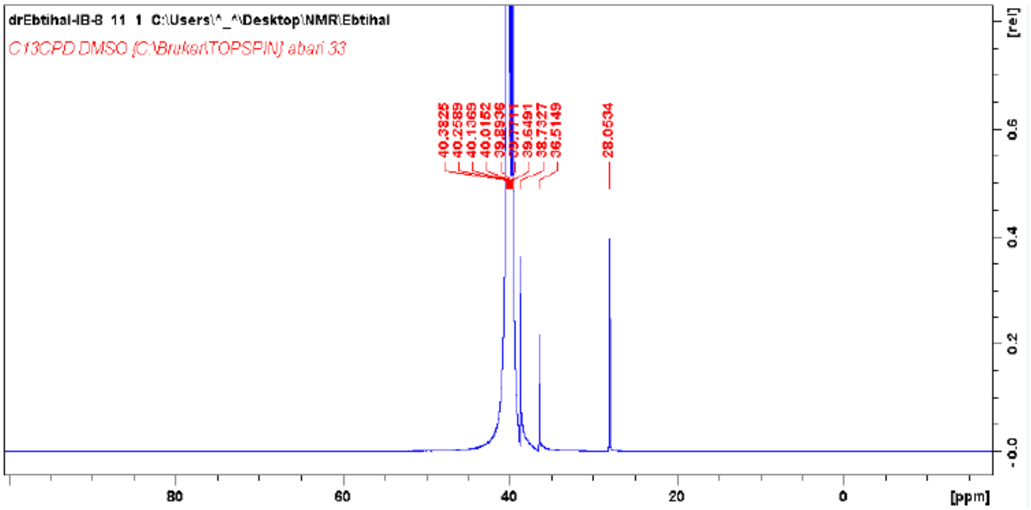

^{1}H NMR Spectra of compound **2** in DMSO-d6.

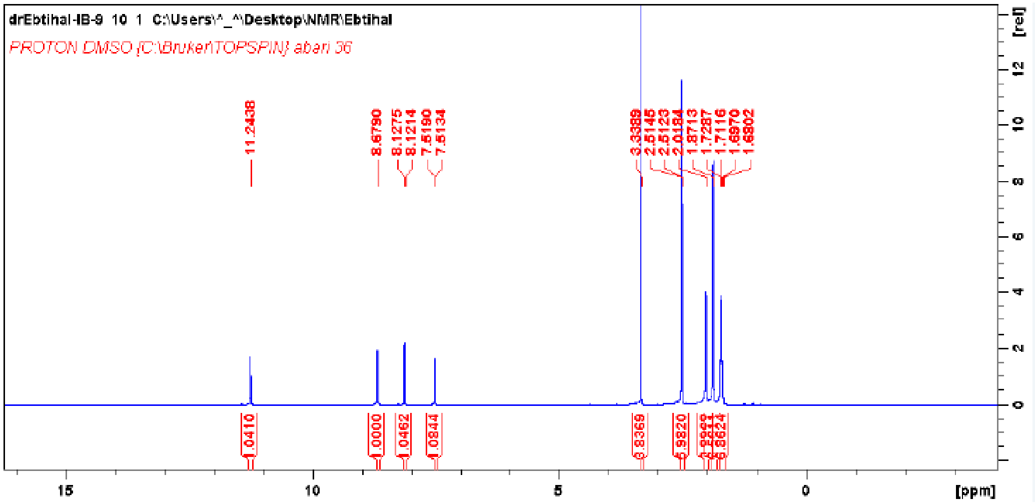

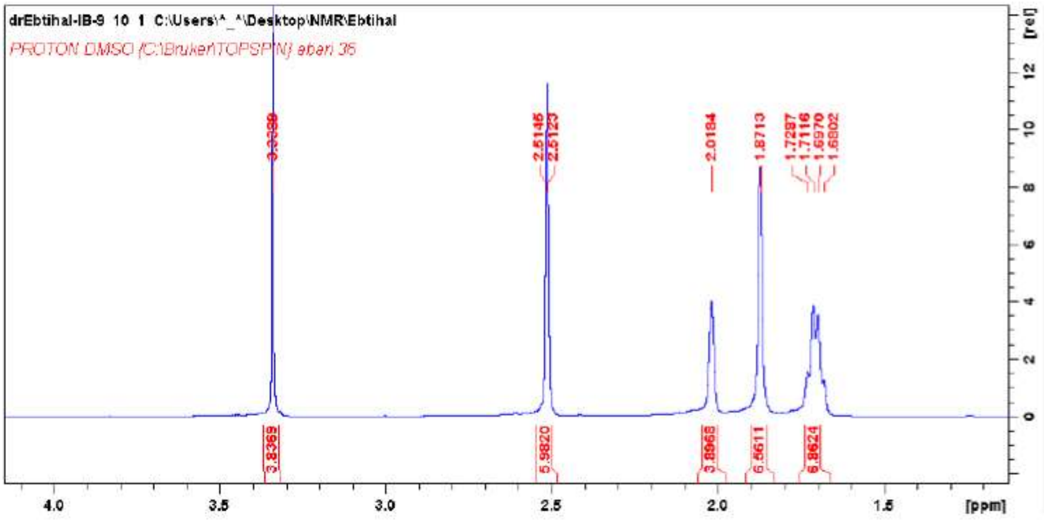

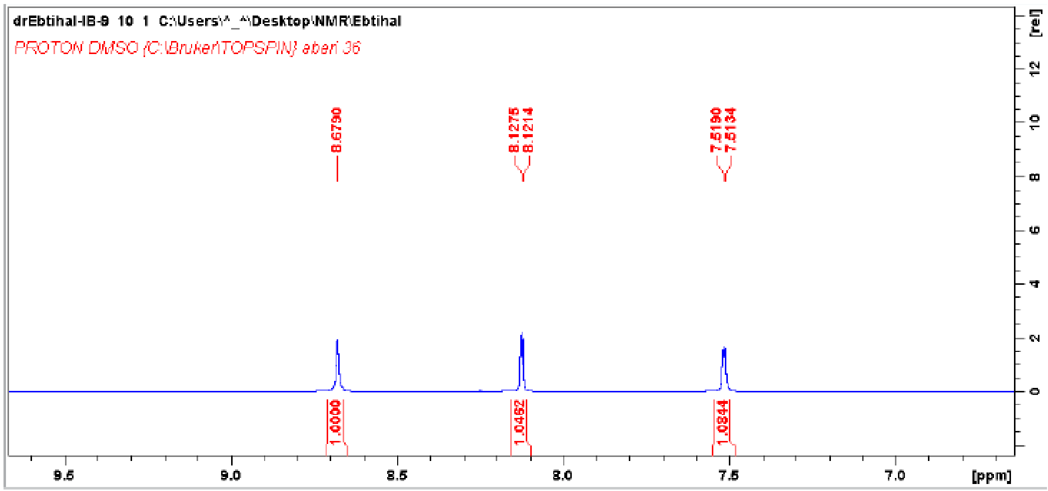

^{13}C NMR Spectra of compound **2** in DMSO-d_6.

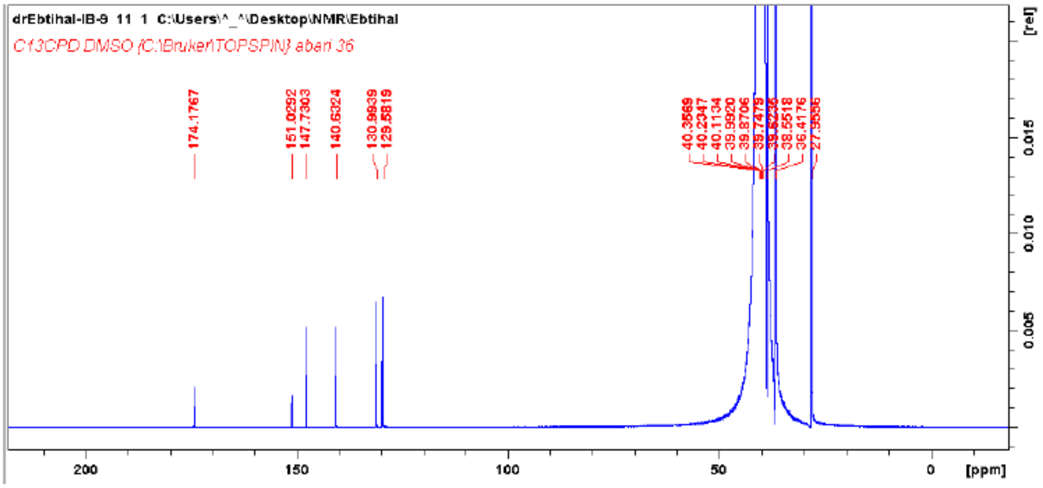

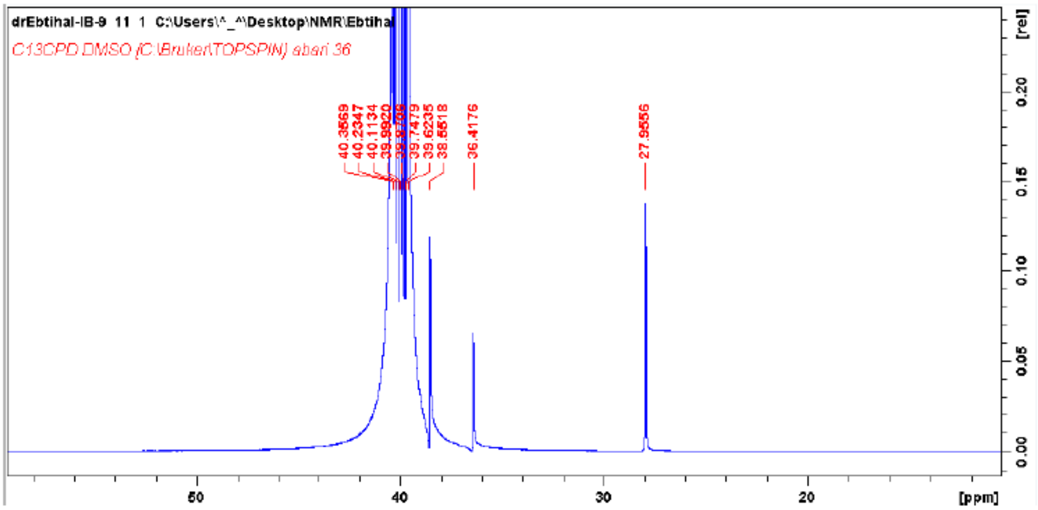

Determination of in vitro antimicrobial activity (agar disc diffusion method)

Sterile filter paper discs (8 mm diameter) were moistened with solution of compounds **1** and **2** in dimethyl sulfoxide of specific concentration (200 µg/disc), and the antibacterial drugs, Gentamicin sulphate and Ampicillin trihydrate (100 µg/disc) and the antifungal drug Clotrimazole (100 µg/disc), were carefully placed on agar culture plates that had been previously inoculated separately with the microorganisms. The plates were incubated at 37 °C, and the diameters of the growth inhibition zones were measured after 24 hours for bacteria and 48 hours for *C. albicans*.

Determination of the minimal inhibitory concentration (MIC)

Compounds **1**, **2**, Gentamicin sulfate, Ampicillin trihydrate, and Clotrimazole were dissolved in dimethyl sulphoxide at a concentration of 128 µg/mL. The two-fold dilutions of the solution were prepared (128, 64, 32, …, 0.25 µg/mL). Suspensions of the microorganisms at concentrations of 106 colony-forming units per mL were inoculated in the corresponding wells. The plates were then incubated at 36°C for 24 hours. The MIC values were determined as the lowest concentrations that completely inhibited visible growth of the microorganism as detected by the unaided eye.
